# Rare primary vasculitis: update on multiple complex diseases and the new kids on the block

**DOI:** 10.1186/s42358-024-00421-8

**Published:** 2024-10-09

**Authors:** Joao Gabriel Dantas, Erika Biegelmeyer, Eduarda Bonelli Zarur, Frederico Augusto Gurgel Pinheiro

**Affiliations:** Rheumatology Division, Universidade Federal de São Paulo, Rua Botucatu, 740, 3° andar, São Paulo, SP 04023-062 Brazil

**Keywords:** VEXAS, Vacuoles, E1 enzyme, X-linked, Autoinflammatory and somatic syndrome, Deficiency of adenosine deaminase 2, ADA2 deficiency, DADA2, VAIHS vasculitis, Autoinflammation, Immunodeficiency, Hematologic defects syndrome, Cogan syndrome, Susac syndrome, Retinocochleocerebral vasculopathy

## Abstract

Systemic vasculitis is a group of rare diseases that share an essential characteristic: inflammation of blood vessel walls. This injury occurs during the disease course, but specific features vary for each entity. In this paper, we will address relevant aspects of the newest monogenic mutation vasculitis, such as deficiency of adenosine deaminase 2 (*ADA2*) and VEXAS syndrome (*UBA1*), and other relevant vasculitis, such as Cogan syndrome and Susac syndrome that may share some similarities with them.

## Introduction

Among the systemic vasculitis group, there are some rarest entities that rheumatologists should consider due to their clinical relevance, independent of their frequency. We are discussing disorders such as the recently discovered monogenic mutation vasculitis, including adenosine deaminase 2 deficiency (DADA2) and vacuoles, E1 enzyme, X-linked, autoinflammatory, somatic (VEXAS) syndrome, and other previously recognized such as Cogan syndrome and Susac syndrome. These diseases are extremely rare but without a correct diagnosis and follow-up, outcomes could lead to relevant morbidity and mortality. Pertinent aspects of each entity are summarized here.

## VEXAS syndrome

**Fig. 1:**
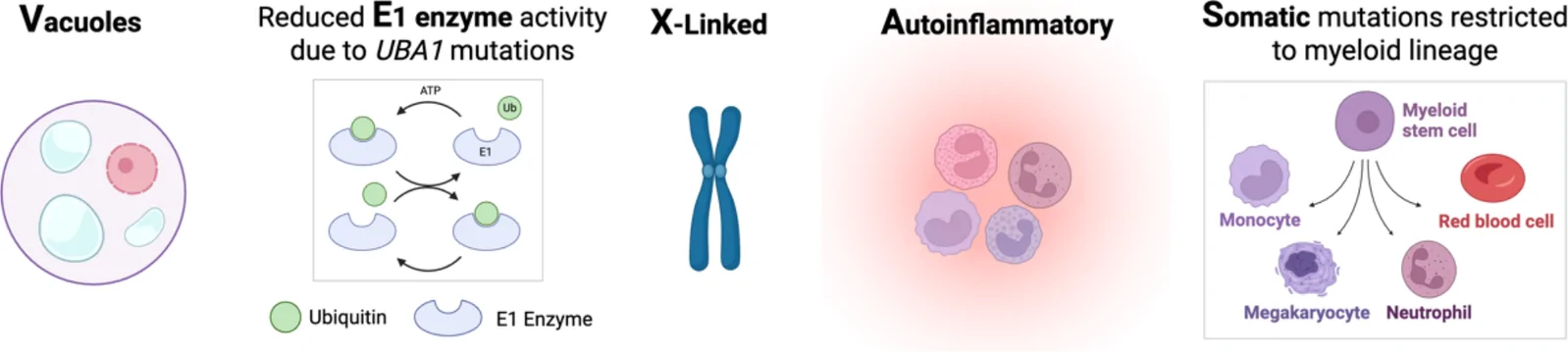
VEXAS acronym

VEXAS syndrome (VS) is a recently described autoinflammatory clinical entity linked to somatic mutations in the *UBA1* (ubiquitin-like modifier activating enzyme 1) gene, first reported in 25 male patients with an adult-onset inflammatory disease with overlapping manifestations and features of hematopoietic dyspoiesis [1]. The acronym VEXAS stands for a group of key abnormalities associated with VS (Fig. 1**)**. As a monogenic disease, all acquired mutations have been identified in the X-linked *UBA1* gene, which encodes the UBA1 enzyme, one of the E1 enzymes, which are responsible for the initiation of all cellular ubiquitylation processes. Ubiquitylation is a post-translational modification that regulates multiple inflammatory pathways, essential for the innate immunity response and disruptions of this process may lead to autoinflammation [2].

The UBA1 enzyme has two isoforms, e.g. UBA1a and UBA1b. Somatic mutations that generate reduced expression of UBA1b are primarily responsible for VS [1]. Mutations clinically expressed as VS were originally described in the start codon 41 (methionine-41), being lineage-restricted to blood myeloid cells, although the original study showed early bone marrow progenitor cells with evidence of mosaicism [1, 3]. Additional mutations in *UBA1* not all involving codon 41 were also described posteriorly [4]. Mutations lead to impaired enzyme activity and reduced intracellular ubiquitylation, a phenomenon closely associated with systemic inflammation and not exclusive of VS [5]. A major immunologic dysfunction takes part in the pathogenesis of the syndrome, consistent with the activation of several innate immunity inflammatory pathways such as interleukin-6, tumor necrosis factor and interferon-γ (IFN-γ) as well as atypical lymphocyte differentiation with increased neutrophil extracellular trap (NET) formation [1].

The *UBA1* somatic mutations leading to VS often appear late in life and predominantly affects men, since it is an X-linked gene [4]. A 2023 exome data study showed presence of disease-causing *UBA1* variants in one in 4269 men older than fifty years [4]. Severe and progressive multisystem inflammation is often the case in VS with a wide spectrum of features [6]. Cartilage, lungs, and skin are primarily involved [7]. Features that combine rheumatologic and hematologic phenotypes are suggestive [7]. The clinical presentation resembles a variety of other causes, imposing a true diagnostic challenge [7]. A whole array of immune-mediated diseases may be mimicked by VS, such as relapsing polychondritis, neutrophilic dermatoses, other primary systemic vasculitis, and systemic lupus erythematosus [1]. Since heterogeneity is the rule, patients may fulfill criteria for some diseases [1].

No manifestation is specific of VS. Recurrent fever is the most common constitutional symptom, with prevalence ranging from 70 to 92% on different cohorts [3, 6, 8, 9]. Weight loss, fatigue and anorexia are also possible. Other common features are macrocytic anemia, thrombocytopenia, skin involvement, nose/ear chondritis, venous thromboembolism and pulmonary involvement [3, 6, 8, 9]. Suspicion relies on the combination of symptoms together with the disease’s systemic nature. Authors suggest that a rational way to consider the diagnosis is the association of multiorgan disease, evidence of systemic inflammation and cytopenia in a 50 year or older male patient [10]. Of note, mimicking rheumatologic conditions is common, and special attention should be given when evaluating refractory cases, especially if progressive hematologic manifestations are present [11]. Retrospective evidence regarding the use of hallmark features of VS showed significant accuracy [6, 12].

Chondritis, sometimes indistinguishable from idiopathic relapsing polychondritis (RP) is frequent. A subset of RP has been recognized in the context of *UBA1* somatic mutations in a RP cohort, defining cases as RP-VEXAS [13]. Patients were all male, had a disease onset in the fifth decade of life or later, presented with ear and/or nose chondritis and hematologic disease [13]. A proposed algorithm stated that, among patients with a defined diagnosis of RP, the combination of male sex plus either a MCV above 100fL or a platelet count below 200.000/µL predicted VS with excellent accuracy [13]. Worse prognosis, fever, skin disease, lung involvement and myelodysplastic syndrome appears to be more prevalent in the RP-VEXAS form of disease [13, 14].

VS almost always presents with varying degrees of bone marrow dysfunction [7, 15]. Anemia is virtually always present and the probability of an elevated MCV is very high [15]. Lymphopenia is also quite common and about half of patients show thrombocytopenia [15]. A distinctive but not specific feature of the syndrome is vacuolization of myeloid and erythroid precursor cells [15, 16]. Hematologic disturbances may lead to hematologic malignancy, with myelodysplastic syndrome being the most frequent condition, varying from 25 to 55%, followed by multiple myeloma, hence the importance of investigating the presence of a monoclonal protein-associated disorder including monoclonal gammopathy of undetermined significance [16]. Hypotheses for the increased risk of hematologic malignancy in VS are the persistent inflammatory state as a promoter of mutant clones and the direct effect of the underlying mutation [17].

The majority of the 25 patients from the original description of VS had pulmonary involvement [1]. A 2023 study with CT scans available from 51 patients showed pleuropulmonary disease in less than 50% of patients, most of them exhibiting ground glass opacities or consolidation [18]. Authors suggested the possibility of underdiagnosis and a low probability of fibrosis [18]. Neutrophilic alveolitis, interstitial pneumonia and pleural inflammation also have been reported [1].

Approximately 90% of patients show skin involvement and cutaneous findings are frequently the first manifestation [19]. Different types of lesions are possible, and no pattern is considered specific [19]. Widespread maculopapular lesions and small nodules is the most common presentation. The histopathological description often shows signs of leukocytoclastic vasculitis or neutrophilic dermatoses such as Sweet’s syndrome [19]. Erythema nodosum-like, urticariform and panniculitis-related lesions are also observed [19].

The spectrum of VS manifestations has gradually increased due to several new reported cases [20]. Other possible features are inflammatory eye disease (uveitis, scleritis, periorbital edema); lymphadenopathy; splenomegaly; interstitial nephritis; abdominal pain, diarrhea; arthralgia and arthritis; myocarditis and pericarditis; aortitis; and peripheral/central nervous system involvement [21].

Blood markers of inflammation are usually high, although unspecific. Bone marrow analysis show vacuolization, hypercellularity, increased myeloid: erythroid ratio and varying degrees of dysplasia [22]. Formal diagnosis is exclusively made upon the detection of compatible pathogenic *UBA1* somatic mutation variants [23]. A 2022 case report shed light on an important issue about exome sequencing [23]. High variant allele frequency can determine misinterpretation of results, with the incorrect notion of hemizygous state [23]. In such cases, additional tissue testing may be necessary to confirm the somatic state linked to VS and not a germline variant of *UBA1* [23]. Tests can be performed in peripheral blood and Sanger technique has been described as the methodology of choice [23].

Optimal therapeutic strategies in VS are still unclear. Management guidance is provided by low quality, limited and heterogeneous data, no randomized controlled trial has been conducted. Treatment is largely based on case series together with the knowledge acquired from treating other autoinflammatory conditions [24]. Pharmacologic approach to treatment is usually dichotomous, relying on controlling the severe inflammation and targeting mutated cells. For the first strategy, glucocorticoids in high doses are effective but tapering is difficult due to recurrence [6]. For patients with complete response under glucocorticoids and low dose required for maintenance, monotherapy may be of choice, although adverse effects of long-term glucocorticoid use are of major concern [11, 21]. For those with poor response or relapsing disease, usually a steroid sparing strategy is initiated, and many drugs have been used in VS [21]. IL-1 blocking agents, tocilizumab and JAK-inhibitors are the main drugs of choice in those cases, with ruxolitinib showing promising results in a retrospective cohort study compared to other JAK-inhibitors [25]. For patients with progressive refractory disease, especially in the context of the hematological aspect of VS, the use azacytidine, a hypomethylating agent, is suggested [26, 27]. Azacytidine have been used in the treatment of hematologic malignancies such as myelodysplastic syndromes and acute myeloid leukemia [3, 6]. Reports showed benefit in the management of VS [26]. For severe cases, bone marrow transplantation is the only potentially curative intervention [28, 29]. Prognosis in VS is closely linked to disease severity and mortality varies among cohorts [6, 8]. A 63% mortality rate in 5 years has been reported, as well as a 10-year median of survival. A clear information about prognosis is still lacking, however, overall prognosis is poor and appears to correlate with hematologic malignant disease [6, 11, 15, 30].

## Deficiency of adenosine deaminase 2 (DADA2)

The DADA2 is a monogenic autoinflammatory disorder with an autosomal recessive inheritance pattern [31, 32]. Since it was first described in 2014, over 600 cases of DADA2 have been reported [31–33]. However, the actual frequency of this disorder is believed to be underestimated [34]. Genetic studies have revealed that mutation variants are carried in the population by at least 1 in 236 individuals. This would indicate a prevalence of 35,000 cases in the population [34], which means that DADA2 might be one of the most common autoinflammatory syndromes.

More than 150 variant mutations of the same gene have been described, according to the INFEVERS database (https://infevers.umai-montpellier.fr) [35]. Biallelic mutation are located in the *ADA2* gene, previously known as *CECR1*, located on chromosome 22q11.1 [35]. Most cases are homozygous with missense mutations (substitutions). Deletion, splicing, and nonsense mutations have also been reported in lower frequency [35].

Initial cases were described on Georgian-Jewish, German, and Turkish patients [31, 32]. Today, the majority of patients are Caucasian (> 75%), with emerging reports in India [36] and Brazil [37]. A genetic study showed a higher allelic carrier percentage in Finland, South Asia, and Latino population. The carrier frequency was lower in African, East Asia, and Ashkenazi Jewish populations [34].

Approximately 70% of cases commence in childhood, nevertheless adult onset is also recognized [33, 38]. Moreover, a significant number of adults likely remain undiagnosed [38]. Prevalence was similar between sexes [39].

Adenosine deaminases (ADA) enzymes regulate purine metabolism by converting adenosine to inosine and deoxyadenosine into deoxyinosine [40]. The two isoforms, ADA1 and ADA2, share partial homology but have distinct physiologic roles [41]. ADA1 is intracellular and expressed in every nucleated cell. In contrast, ADA2 is primarily expressed by myeloid cells and secreted to the extracellular space, mainly in inflammatory scenarios [40, 41].

Besides participating in purine metabolism, ADA2 also works as a growth factor, playing a role in the differentiation and activation of immune cells [40]. Furthermore, ADA2 is believed to contribute to the maintenance of vascular integrity [42].

Even though DADA2 pathogenesis is not entirely understood, one of the hallmarks is the polarization of monocyte differentiation towards the macrophage M1 subtype [43]. This results in a pro-inflammatory environment, with increased release of TNF-α, IL-1β, and IL-6, which propagate inflammatory response and endothelial damage [31]. Furthermore, DADA2 upregulates IFN-γ signaling and triggers chronic neutrophile activation with spontaneous NET formation [42, 43]. DADA2 also impair interactions between T and B cells [44].

**Fig. 2:**
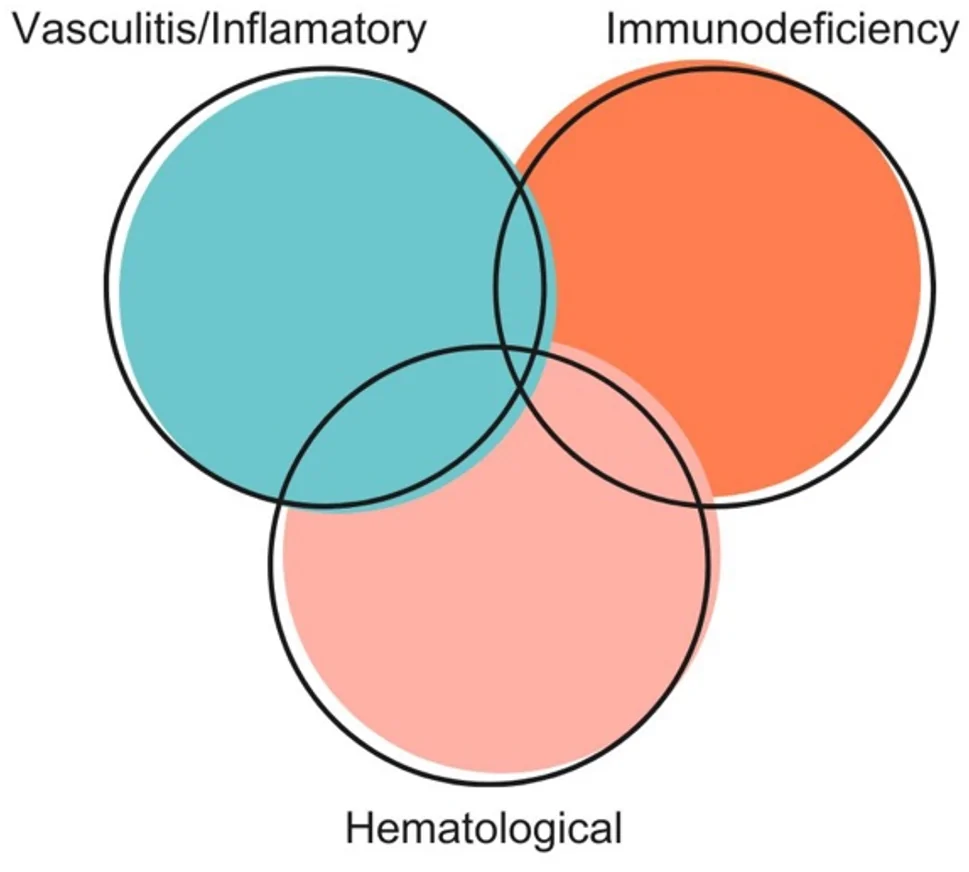
Deficiency of adenosine deaminase 2 (DADA2) clinical manifestations

The first described cases of DADA2 were characterized by a polyarteritis nodosa (PAN)-like cutaneous vasculopathy coupled with early-onset stroke in young children [31, 32]. Further research expanded the clinical spectrum, that is categorized into three groups: (I) inflammatory/vasculitis, (II) hematologic manifestations, and (III) immunodeficiency (Fig. 2**)** [45]. Patients usually exhibit a dominant clinical phenotype, although overlapping is frequent. Hematological and immunodeficiency manifestations rarely occur in isolation [39, 45].

Inflammatory/vasculitis phenotype is the most common [33, 37–39, 46]. Cutaneous and neurological symptoms are predominant in DADA2 patients, differently from PAN [39]. Cutaneous involvement occurs in about 70–90% of cases, usually with livedo racemosa (47–74%) [33, 39]. PAN-like nodules (23–57%), Raynaud’s phenomena, acral digital gangrene, and erythema nodosum can also appear [33, 37, 39].

Skin biopsy resembles PAN histopathology, with medium-size vasculitis in the deep dermis, with dense transmural infiltrate of neutrophils with karyorrhexis [39, 47]. Fibrinoid necrosis and chronic surrounding inflammation may be present [39]. Leukocytoclastic vasculitis of small vessels, as well as vasculopathy with lymphovascular occlusion and intravascular thrombi, can also be found [31, 32, 36, 39].

The typical neurological manifestation is transient ischemic attack (TIA)/ischemic stroke (41–43%) [33, 39, 46, 48]. Strokes are usually lacunar, affecting mostly basal ganglia, brain stem, internal capsule, and cerebellum [33, 39, 46, 48]. In a 60-patient cohort, the mean age for the first stroke was 6.5 years, with an average of 3 strokes per patient [39]. Additionally, stroke was the disease presenting symptom in several patients, and about half of them experienced their first stroke before reaching five years old [39, 48]. Intracerebral hemorrhage may occur less frequently, typically after an ischemic stroke and/or with the use of aspirin or anticoagulants [31, 32, 39, 48].

Vasculitic DADA2 presentation may also resemble Sneddon Syndrome, with ischemic stroke, livedo racemosa, and biopsy with intravascular thrombi. In addition, DADA2 may present positive antiphospholipid (aPL) antibodies in 27% of patients [48]. Because the treatments for these two conditions are considerably distinct, there should be caution when diagnosing Sneddon Syndrome without performing ADA2 screening.

Other neurologic manifestations include polyneuropathy, mononeuritis multiplex, cranial nerve palsies, spastic paraplegia, sensorineural hearing loss, and posterior reversible encephalopathy syndrome (PRESS) [37, 42, 49]. Ophthalmologic involvement may occur as optic neuritis, uveitis, retinal artery occlusion, and orbital pseudotumor [42, 49].

Vasculopathy may affect other organs, such as intestinal ischemia, hepatomegaly, hepatic fibrosis, portal hypertension, mesenteric and renal aneurysms, testicular pain, cardiomyopathy, inflammatory myositis, and other manifestations [33, 39, 46]. Also, unspecific symptoms such as musculoskeletal manifestations and periodic fever may occur in more than half of patients [39, 46].

Hypogammaglobulinemia with low immunoglobulin M (IgM) is the most common presentation in the immunodeficiency phenotype [31, 50]. Low immunoglobulin G (IgG), generalized lymphadenopathy, and splenomegaly may also occur [51]. Recurrent infections, typically caused by bacteria, herpes zoster, and verrucous warts, have been reported [39, 51]. Immunodeficiency is generally milder when associated with the vasculitic form of disease than the hematologic DADA2 phenotype [39]. Severe presentations, such as combined variable immunodeficiency disease, autoimmune lymphoproliferative syndrome (ALPS), and lymphoma, may occur more rarely [33, 39].

Finally, the hematological phenotype is the least frequent, often more severe, and predominantly affects younger patients [50]. Lymphocytopenia is the most common hematological feature [50]. Anemia is usually multifactorial and may appear in half of the patients [39, 50]. Coombs-positive autoimmune hemolytic anemia has been described [36]. Bone marrow evaluations can reveal lineage hypoplasia, and severe conditions such as Diamond-Blackfan anemia, pure red cell aplasia, and bone marrow failure more rarely occur [50].

While patients with vasculitis/systemic inflammation usually present up to 3% of residual activity of ADA2, patients with hematological manifestation present minimal or absent ADA2 activity [49, 52]. There is a genotype-phenotype correlation, where the vasculitis manifestations are more frequently associated with *ADA2* missense mutations, whereas hematological phenotypes have more catalytic mutations, such as nonsense, insertions, and deletion variants [49, 52].

In a comparative study of adult versus child disease-onset, adults presented more cutaneous nodules, ulcers, purpura, peripheral neuropathy, gastrointestinal events, and infections associated with humoral deficiency [38]. Whereas children exhibited more strokes, anemia, and neutropenia [38]. Also, adult-onset disease correlated with *ADA2* hypomorphic mutations [38].

Despite the genotype-phenotype correlation, there is evidence that epigenetics and environment influence the clinical presentation. Studies with siblings with same mutations showed differences in severity and age of onset [53, 54].

DADA2 is a potentially fatal disease, with a mortality rate of 8% before 30 years old [55]. Worst outcomes are associated with recurrent stroke, gastrointestinal ischemia, recurrent infections, and hematological phenotype [39].

Diagnosis is established in symptomatic patients who present low plasma ADA2 enzymatic activity or display a compatible genetic sequencing of the *ADA2* gene [45]. A recent consensus for managing DADA2 recommends that both methods should be employed when feasible since both have limitations [45].

Normal ADA2 activity rules out the condition; conversely, low ADA2 levels supports the diagnosis [45]. ADA2 activity levels can be assessed using spectrometry or high-performance liquid chromatography assay [45]. The availability of the tests is usually limited to research [45].

Genetic diagnosis is also established by the identification of biallelic pathogenic or likely pathogenic *ADA2* variants [56]. *ADA2* genetic testing may be found in most commercial sequencing panels for inborn errors of immunity or autoinflammatory syndromes [45].

When *ADA2* variants of unknown significance (VUS) are detected, the assessment of ADA2 enzyme activity is recommended [45]. In addition, when clinical suspicion of DADA2 remains, despite negative genetic testing, it is suggested that further evaluation be performed for intronic, splicing, and gene structural variants that may not be identified on standard genetic techniques [45, 57].

When a patient is diagnosed, all siblings should be screened [45]. Any relative with suspected symptoms should also be considered for screening. Individuals are considered carriers when only monoallelic *ADA2* variants are detected. Most carriers are asymptomatic; however, some may present mild features of DADA2 [44]. Asymptomatic individuals with biallelic pathogenic mutations are considered pre-symptomatic patients and there is ongoing debate regarding their clinical progression [45].

The 2023 consensus from DADA2 experts recommends ophthalmologic evaluation and magnetic resonance imaging/angiography (MRI/MRA) brain scan for all patients [45]. The DADA2 disease activity and disease damage score have been proposed to standardize assessment [58]. Early diagnosis and treatment are crucial considering the unfavorable prognosis. Treatment is based on retrospective studies, case series, and expert opinion [45].

For the vasculitic/inflammatory phenotype, Tumor Necrosis Factor inhibitors (TNFi) are the preferred intervention, since they are the only medications that prevented recurrent strokes [48]. Therapy should be lifelong; recurrence was observed following suspension. Consensus suggests association with disease-modifying antirheumatic drugs to mitigate anti-drug antibody formation, though this risk remains uncertain [39, 45]. Anti-drug antibody screening is warranted in the event of a flare. In refractory cases, biologics targeting IL-1 and IL-6 have been reported [59].

For acute stroke, glucocorticoid and/or TNFi should be initiated [45]. The benefit of antiaggregant, anticoagulant, or antithrombotic medications in these patients is unclear and is not routinely suggested because of the risk of hemorrhagic transformation [45].

The hematological phenotype is refractory to TNFi and glucocorticoids. Allogeneic hematopoietic stem cell transplantation (HSCT) is curative for severe and refractory cases, such as bone marrow failure [49, 52].

Patients with immunodeficiency may need prophylactic antibiotics and intravenous immunoglobulins (IVIG) in cases of recurrent infections and humoral deficiency. Immunizations are preconized in all patients [49, 52].

## Cogan syndrome

Cogan syndrome (CS) is a rare chronic inflammatory disease classified as a variable vessel vasculitis [60] that occurs especially in young Caucasian adults of both sexes [61–63]. The first description as a specific entity occurred in 1945, by the ophthalmologist David G. Cogan, as a syndrome of nonsyphilitic interstitial keratitis (IK) and vestibuloauditory symptoms [64]. The most common clinical presentations of CS are IK and Ménière-like episodes [62].

IK is a classic eye presentation that manifests as eye redness, pain, photophobia, and blurred vision [62] and is usually bilateral [63]. Other eye structures can be inflamed, resulting in iridocyclitis, conjunctivitis, episcleritis, anterior or posterior uveitis, and retinal vasculitis [62]. The worst outcome, blindness, is reported in 8% of patients [65].

**Fig. 3:**
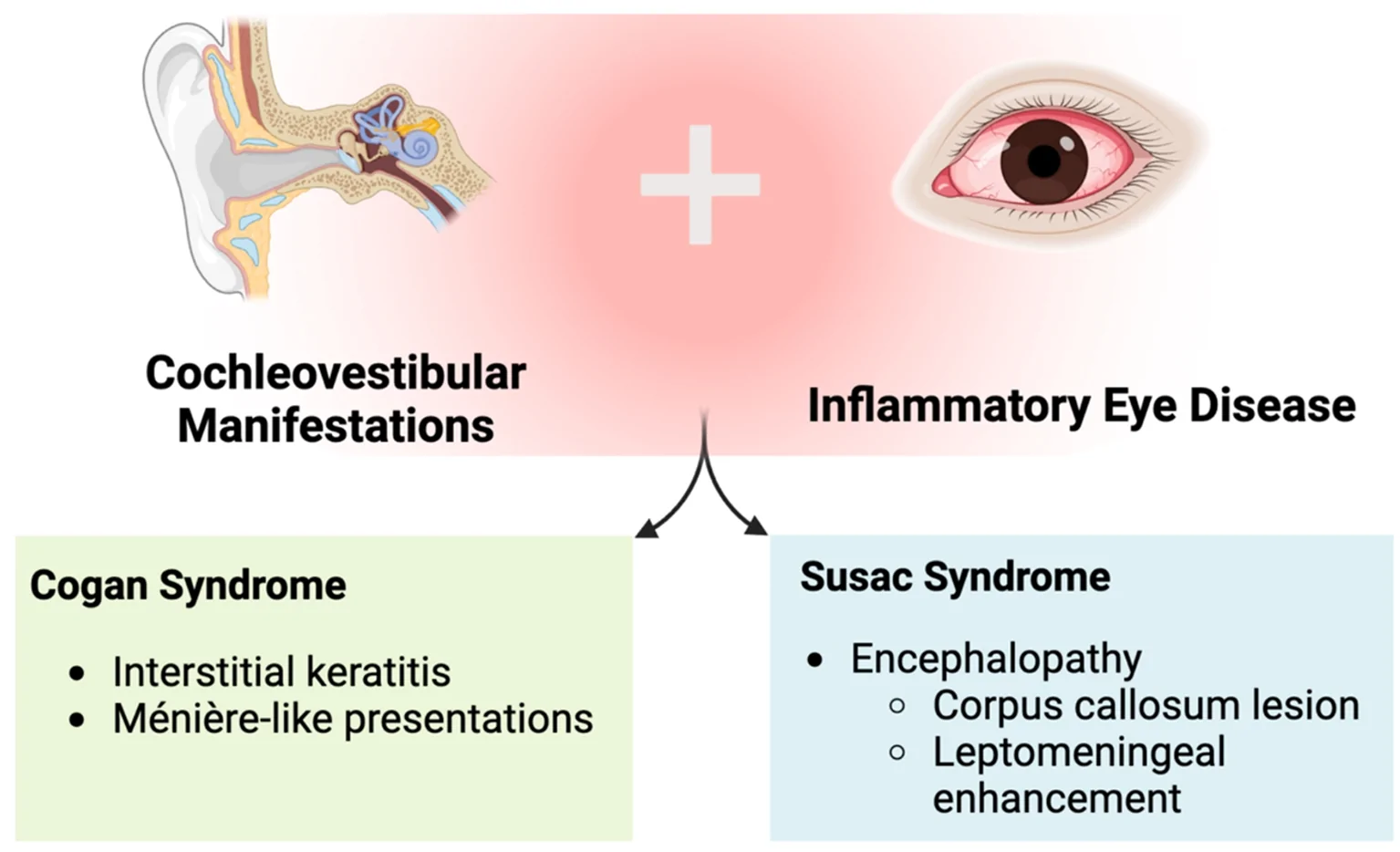
Main manifestations of Cogan syndrome and Susac syndrome

Regarding cochleovestibular manifestations, CS generally has a Ménière-like presentation, with abrupt onset of vertigo, ataxia, nausea, vomiting, and tinnitus, generally associated with severe and bilateral hearing loss (Fig. 3) [63]. Audiometry has a sensorineural pattern, preferentially affecting low- and high-range frequencies, similar to the findings in Ménière disease [62]. In contrast to visual outcomes, the loss of hearing associated with CS is usually severe, with permanent bilateral deafness occurring in 43.5% of patients, usually 3 months after the onset of initial symptoms [63].

Vasculitic manifestations occur in 15–21% of patients [62] and may include arteritis (of any size), aortitis, aortic aneurysms, and aortic or mitral valvulitis [60].

The diagnosis of CS is defined by the association of visual and cochleovestibular abnormalities after the exclusion of alternative infections (syphilis, tuberculosis, chlamydial infection) and inflammatory diseases (sarcoidosis, PAN, granulomatosis with polyangiitis and Takayasu arteritis) [62].

Treatment traditionally consists of systemic corticosteroids and/or immunosuppressive agents, such as methotrexate, azathioprine, leflunomide, mycophenolate, cyclophosphamide, cyclosporin, TNFi, rituximab (anti-CD20), or tocilizumab (anti-IL6 receptor) [62, 65]. The best treatment option is related to clinical severity [62].

## Susac syndrome

Susac syndrome (SuS) is another rare disease characterized by the triad of hearing impairment, visual disturbance, and encephalopathy. In 1979, SuS was first described by Dr. John O. Susac in two patients with “personality changes and paranoid psychosis” associated with retinal artery occlusions [66]. Its incidence ranges from 0.024 to 1.2/100,000 inhabitants [66, 67]. SuS mainly affects young women, with a mean age of onset of 31.6 years (± 10.4, 8–65 years) and a male to female ratio of 1:3.5 [67].

The pathophysiology is unclear but is thought to be mediated by CD8^+^ T cells resulting in endothelial damage to small vessels of the brain, inner ear, and retina [67].

The classical triad occurs in only 13% of patients at diagnosis, resulting in an additional challenge for diagnosis [67]. The diagnosis is based on the presence of typical features of the brain, eye, and ear.

Neurological manifestations are the most common characteristic at diagnosis, with subacute encephalopathy (75% of cases) with varying degrees of involvement, ranging from impairment in attention and executive functions to severe confusional status or psychiatric manifestations [66]. Cerebrospinal fluid evaluation may be normal or altered, with elevated protein levels (80% of patients) and lymphocytic pleocytosis in 50% of patients [66]. The presence of oligoclonal bands can occur in 15% of cases [66].

The presence of small rounded white matter lesions on T2- and T2-FLAIR-weighted brain MRI, mostly supratentorial, is present in all patients with central nervous system involvement [66]. The evidence of a “string of pearls” at the internal capsule is a very important finding for SuS. Another important aspect of SuS, considered pathognomonic, is the association between microvascular ischemic lesions in the corpus callosum and leptomeningeal enhancement (Fig. 3) [66].

Ocular involvement is a result of multiple retinal artery occlusions, which generally spare the macular region [66]. Retinal fluorescein angiography is the best tool for revealing retinal artery occlusion [66].

The cochleovestibular manifestations are composed of sensorineural hearing loss (up to 90% of cases), with abrupt onset that is frequently bilateral and is associated with tinnitus or intense rotatory vertigo. Audiometry usually shows hearing loss predominating at low to midtone frequencies, suggesting damage to the apical part of the cochlea [66].

The treatment of SuS is empirical and based on the hypothesis of inflammatory microvasculopathy, supporting the use of immunosuppressive and immunomodulatory agents [66]. The cornerstone is corticosteroid (high-dose) and, for severe cases, the additional use of immunosuppressive agents, such as cyclophosphamide, azathioprine, mycophenolate mofetil or rituximab [66]. Anti-platelet drugs are often used as an add-on therapy [66]. Other possible agent is IVIG (for patients with corticosteroid single use and active disease). Other anecdotally reported options are infliximab, adalimumab, plasma exchange, cyclosporine, and hematopoietic stem cell transplantation [66].

## Conclusion

These entities have a considerable overlap of manifestations and physician’s awareness and knowledge are essential to recognize each of them. Despite being rare diseases, outcomes may be devastating.

## Data Availability

Data sharing is not applicable to this article as no datasets were generated or analyzed during the current study.

## Abbreviations

ADA2: Adenosine deaminase 2
ALPS: Autoimmune lymphoproliferative syndrome
aPL: Antiphospholipid
CS: Cogan syndrome
DADA2: Deficiency of adenosine deaminase 2
FLAIR: Fluid attenuated inversion recovery
IgG: Immunoglobulin G
IgM: Immunoglobulin M
IK: Interstitial keratitis
IFN-γ: Interferon-γ
IVIG: Intravenous immunoglobulin
JAK: Janus kinase
MCV: Mean corpuscular volume
MRA: Magnetic resonance angiography
MRI: Magnetic resonance imaging
NET: Neutrophil Extracellular Traps
PAN: Polyarteritis nodosa
PRESS: Posterior reversible encephalopathy syndrome
RP: Relapsing polychondritis
SuS: Susac syndrome
TIA: Transient ischemic attack
TNFi: Tumor Necrosis Factor inhibitors
UBA1: Ubiquitin-like modifier activating enzyme 1
VEXAS: Vacuoles, E1 enzyme, X-linked, autoinflammatory, and somatic syndrome
VUS: Variants of unknown significance
VS: VEXAS syndrome
